# Survey Responses of School Closures During the COVID-19 Outbreak in Taiwan

**DOI:** 10.3389/fpubh.2022.726924

**Published:** 2022-03-16

**Authors:** Kuo-Yu Chao, Tung-Yuan Hsiao, Wei Cheng

**Affiliations:** ^1^Department of Nursing, Chang Gung University of Science and Technology, Taoyuan, Taiwan; ^2^Division of Colon and Rectal Surgery, Chang Gung Memorial Hospital–Linkou, Taoyuan, Taiwan; ^3^National Alliance of Parents Organization, Taipei, Taiwan; ^4^Department of Pathology, Kee-Lung Hospital, Ministry of Health and Welfare, Kee-Lung, Taiwan; ^5^School of Nursing, National Taipei University of Nursing and Health Sciences, Taipei, Taiwan; ^6^Department of Nursing, Ching Kuo Institute of Management and Health, Kee-Lung, Taiwan

**Keywords:** school closures, survey, COVID-19, parents, outbreak

## Abstract

**Background:**

Taiwan faced a surge of COVID-19 infections in May 2021. Because new cases were quickly increasing, parents called for school closures. A national parent group used an online survey to collect opinions about upcoming school closings planned by the Ministry of Education. This study evaluated the results of the survey for all respondents and investigated the level of viral transmission following school closures among students in Taiwan.

**Methods:**

An online survey titled “Survey of Opinions of School Closures during the Current COVID-19 Outbreak” (SOSC-COVID-19) was designed by the national parent association and then distributed to members of the community throughout Taiwan *via* local parent groups from May 17 to 18, 2021. The survey included an open-ended respondents' opinions about school closures. Differences among regions and socioeconomic scores (SES) were analyzed with chi-square tests.

**Results:**

A total of 8,703 completed survey forms data were analyzed. Nearly all respondents (7,973, 91.6%) approved of school closures; there were no differences of opinions inside and outside municipalities or by regional SES scores. Only 8.4% of respondents were opposed to any type of school closure, believing parents should decide whether their child attended school, which also did not vary with region or SES score. Qualitative feedback from parent and teacher responders indicated students' health and economic impacts were additional concerns that influenced their choice of whether the government or parents should decide about school closures. On the afternoon of May 18, 2021, the government of Taiwan closed all schools. Although a spike in new cases of COVID-19 occurred among students 10 days after school closures, over the next 40 days new cases declined, falling to zero by July 5th.

**Conclusions:**

Despite the inability of nationwide school closures to completely halt transmission of the virus within families during the COVID-19 outbreak, school closures helped to impede transmission between students.

## Introduction

On March 12, 2020, the World Health Organization (WHO) declared severe acute respiratory syndrome caused by the virus known as SARS-CoV-2 to be a pandemic (1). Many countries attempted to control this pandemic disease, now referred to as COVID-19, by imposing nationwide school closures, which several countries continue to enforce. School closures can be a useful intervention during a pandemic, based on experiences with influenza (2). However, no data are available on the effectiveness of school closures specifically because they were part of a broad range of quarantine and social distancing measures to reduce the spread of COVID-19. Studies have concluded that the combination of quarantine and social distancing was effective in controlling the epidemic in mainland China (3) and Hong Kong (4), but the relative contribution of school closures was not assessed.

Taiwan has Mostly been spared from the impact of COVID-19 infections with an infection rate of <10 cases per week since the beginning of the pandemic in 2020. The alpha variant of SARS-CoV-2 in Taiwan was first reported for two cases on December 31, 2021. Then, on April 20, 2021, a small outbreak occurred, which became worse during the week of May 17, 2021, with a surge of new cases, most were the alpha variant. The infection rate increased to more than 900 cases per week, during which time the COVID-19 vaccination rate was only 0.93 per 100 people (5). Most of the cases were centered in Taipei and New Taipei; therefore, the mayors of these cities announced the closing of all kindergartens, elementary schools, and junior and senior high schools on May 17 and most universities also closed.

However, parents elsewhere in the country were also concerned about the rising number of cases. Although local areas had the option to close schools, there were no nationwide criteria. To determine if parents would support a decision by the Ministry of Education (MOE) to close schools nationwide, a national parent group designed a survey to collect parents' opinions of school closures, which was distributed on May 17, 2021.

The primary aim of this study was to determine if there were regional differences in parents' opinions regarding school closures during the outbreak of COVID-19 in May 2021. This study also investigated the effects of the school closures in reducing further viral transmission among students. The parental opinions of school closures and the effect of school closures on transmission of COVID-19 in Taiwan could be used to guide school systems in other countries, especially as outbreaks of the new variants of the virus occur.

## Methods

### Design

The SOSC-COVID-19 was a cross-sectional survey sent to regional parent organizations in 20 districts throughout Taiwan. Regional leaders promoted the survey through social media, which provided a link to the online survey. The link was available to anyone in the community and was active between 5:00 p.m. on May 17, 2021, and 10:00 a.m. on May 18, 2021.

### Participants

The internet survey was dependent upon a convenience sample of participants to gather opinions of parents with school-aged children, and other individuals in Taiwan about school closures. In Taiwan, 85–90% people over 16 years-old have access to mobile phones and the Internet (6). The only inclusion criteria were access to the Internet *via* a smartphone or computer. Because responding to the survey required the ability to read, those who were unable to read Chinese were unable to participate.

### Ethical Considerations

This study was approved by the Ethics Committee of Taipei Hospital, Ministry of Health and Welfare (TH-IRB-0021-0017). All procedures were in accordance with the ethical standards of this committee and the 1964 Helsinki declaration and its later amendments or comparable ethical standards.

### The Survey

**Figure 1:**
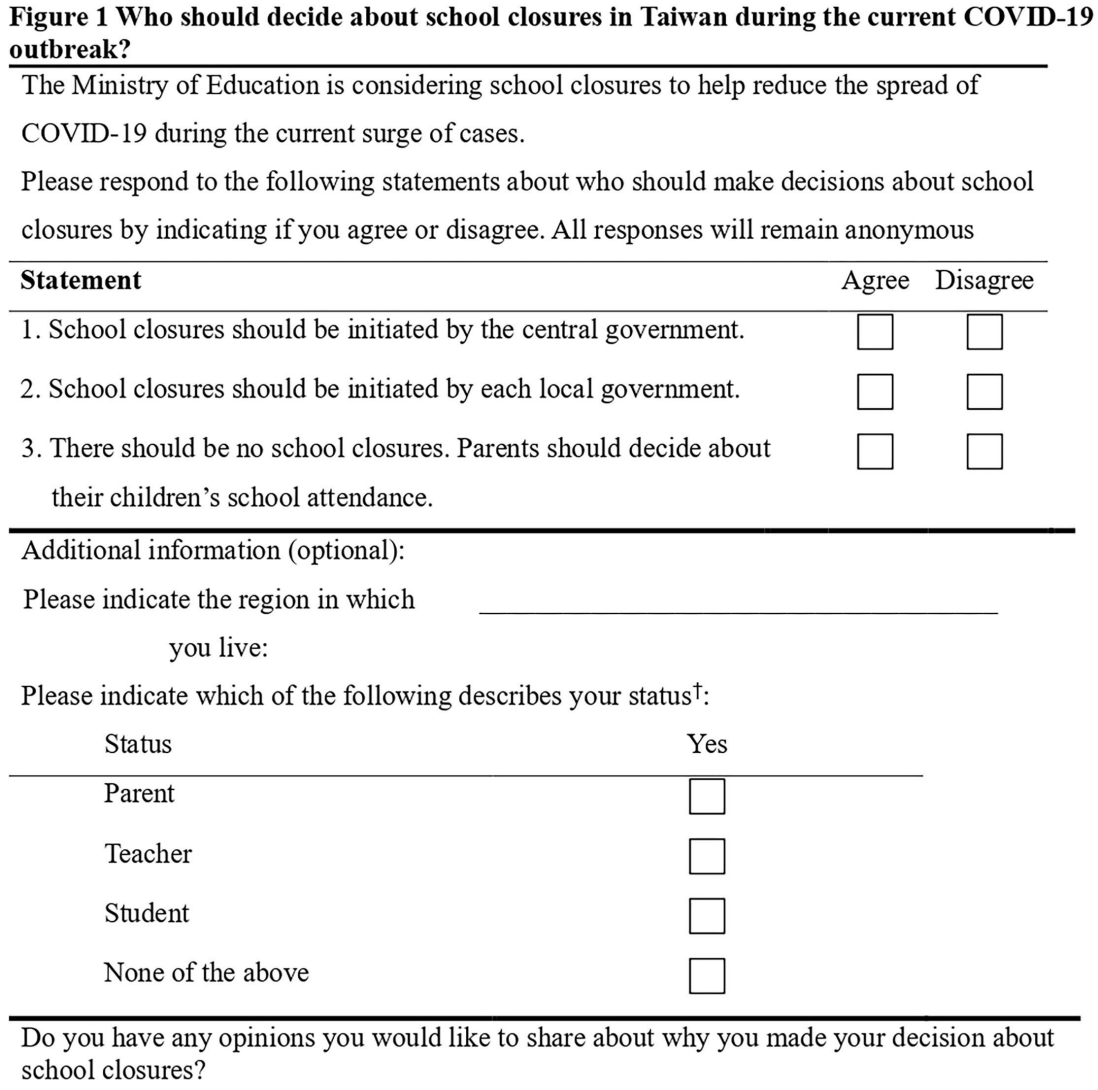
The survey designed by the National Alliance of Parents Organization in Taiwan, “Survey of Opinions of School Closures during the Current COVID-19 Outbreak” (SOSC-COVID-19). ^†^If participants selected “parents” as well as “teacher” or “student”, they were given the status of parent.

**Figure 2:**
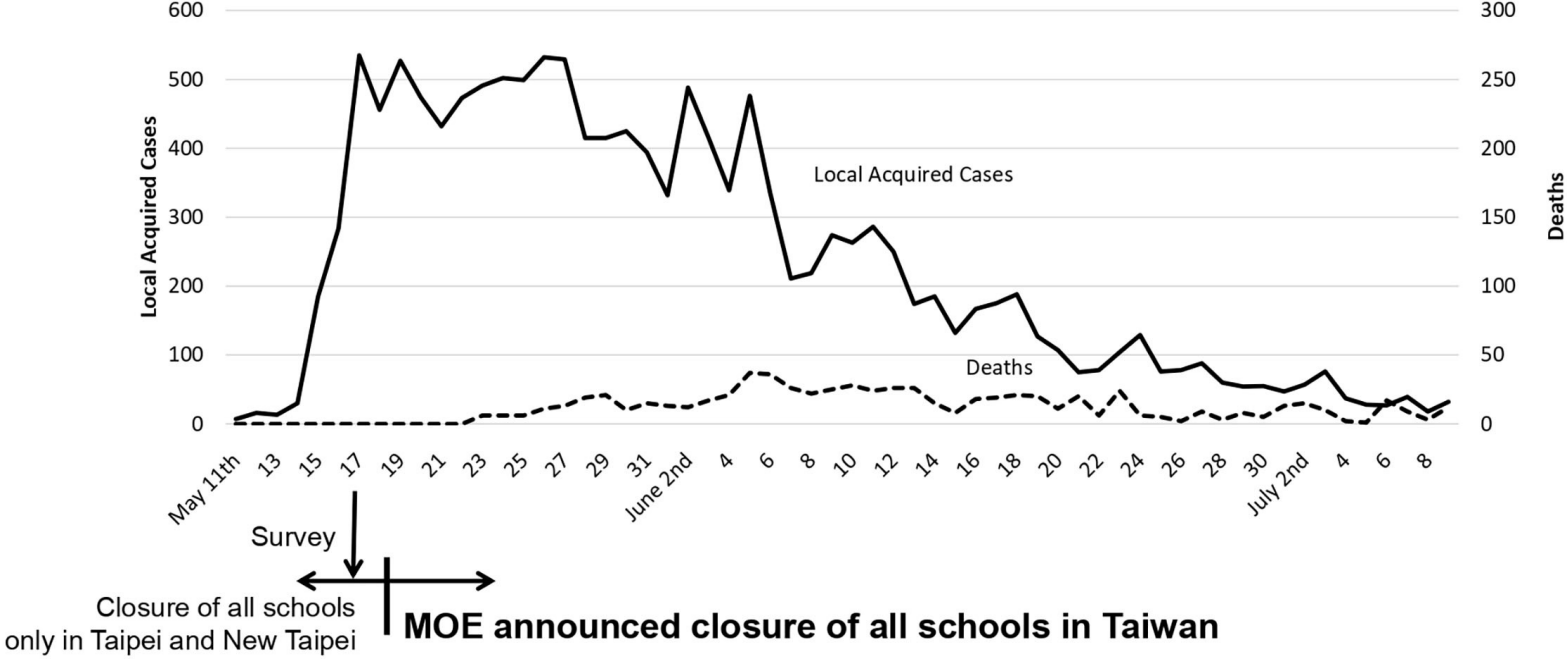
Number of new daily local acquired COVID-19 cases and the timing of the survey of SOSC-19. Data excludes 1–10 imported cases per day (Ministry of Health and Welfare).

The National Alliance of Parents Organization in Taiwan developed an online survey, titled “Survey of Opinions of School Closures during the Current COVID-19 Outbreak” (SOSC-COVID-19), which was designed on May 17, 2021, and asked the question, “Who should decide about school closures in Taiwan during the current COVID-19 outbreak?” (Figure 1). This was a critical time point at which a record high of 333 new COVID-19 cases were reported (which were corrected to 535 cases later). National Alliance of Parents Organization disseminated the SOSC-COVID-19 from May 17 to 18, intending to unofficially send the survey results to the central government (Figure 2). Respondents also had the option of indicating if they were a parent, teacher, student, or other. In addition, they had the option of responding to an open-ended question, “Do you have any opinions you would like to share about why you made your decision about school closures?”

### School Closures and Viral Transmission Among Students

To investigate the effects of the school closures in reducing further viral transmission among students, data were obtained for locally acquired new cases of COVID-19 for students in Taiwan between May 11 and July 9, 2021. These data were available from the Ministry of Education of Taiwan.

### Data Analysis

Quantitative data were analyzed using SPSS version 28.0 for Windows (Armonk, NY: IBM Corp). Descriptive statistics were used for frequency (*n*, %). Chi-square tests assessed the differences between participants' opinions. Statistical significance was set to *p* < 0.05 for all statistical comparisons.

Because the survey only asked who should decide school closures, qualitative data were collected about what influenced the choice made by the responders. The use of open-ended questions provides data that is more diverse than is possible with a forced response as respondents have an opportunity to offer more authentic opinions (7). The authors read through the opinions and sorted them according to support for government school closures or support for parental choice. Opinions were read and categorized by weighting which opinions most frequently fell into a category, which was based on the reason given for their decision.

## Results

### Participant Characteristics

A total of 8,712 participants filled in the online SOSC-COVID-19 survey from May 17 to 18, 2021. However, three surveys were incomplete, and six respondents completed the survey more than once. Thus, data were analyzed for 8,703 participants. The sample loss rate was 0.1%.

The geographical and economic distribution of respondents to the survey is shown in Table 1. The largest groups of respondents were from the inner municipalities of Taichung City (*n* = 2,013, 23.1%), Taoyuan City (*n* = 1,352, 15.5%) and Kaohsiung City (*n* =1,173, 13.5%) and the outer municipality of Changua County (*n* = 1,774, 20.4%). The two cities where school closures were already announced (Taipei City and New Taipei), are inner municipalities and only a small number of surveys were received (*n* = 150, 1.7% and *n* = 169, 1.9%, respectively).

**Table 1:** Geographic and economic distribution of respondents to the online SOSC-COVID-19 survey in Taiwan (*N* = 8,703).

**Region**	**SES score^**a**^**	***n* (%)**
**Inner municipality**
Taichung city	> 41	2,013 (23.1%)
Taoyuan city	> 41	1,352 (15.5%)
Kaohsiung city	> 41	1,173 (13.5%)
Tainan city	> 41	246 (2.8%)
New taipei city	> 41	169 (1.9%)
Taipei city	> 41	150 (1.7%)
**Outer municipality**
Changhua county	>41	1,774 (20.4%)
Hsinchu county/city	>41	734 (8.4%)
Chiayi county/city	>41	114 (1.3%)
Penghu, lianjiang and kinmen county	>41	88 (1.0%)
Yilan county	>41	73 (0.8%)
Miaoli county	>41	67 (0.8%)
Keelung city	>41	39 (0.4%)
Pingtung county	≤ 40	486 (5.6%)
Nantou county	≤ 40	88 (1.0%)
Yunlin county	≤ 40	78 (0.9%)
Taitung county	≤ 40	31 (0.4%)
Hualien county	≤ 40	28 (0.3%)

*SES, socioeconomic status*.

a *SES ≤ 40 = income <NT800,000/USD 28,500 per family annually and less employment opportunity*.

The socioeconomic status (SES) scores reported by the National Development Council were calculated by the incomes and employment opportunities for each region (8) (see Table 1). A total of 7,992 participants (91.8%) lived in areas with SES scores above 40. All inner municipalities have SES scores >41. A total of 711 participants (8.2%) lived in areas with SES scores ≤ 40 (with income <NT800,000/USD 28,500 per family annually and less employment opportunity); these respondents represented 19.8% of the outer municipalities.

### Quantitative Survey Results

**Figure 3:**
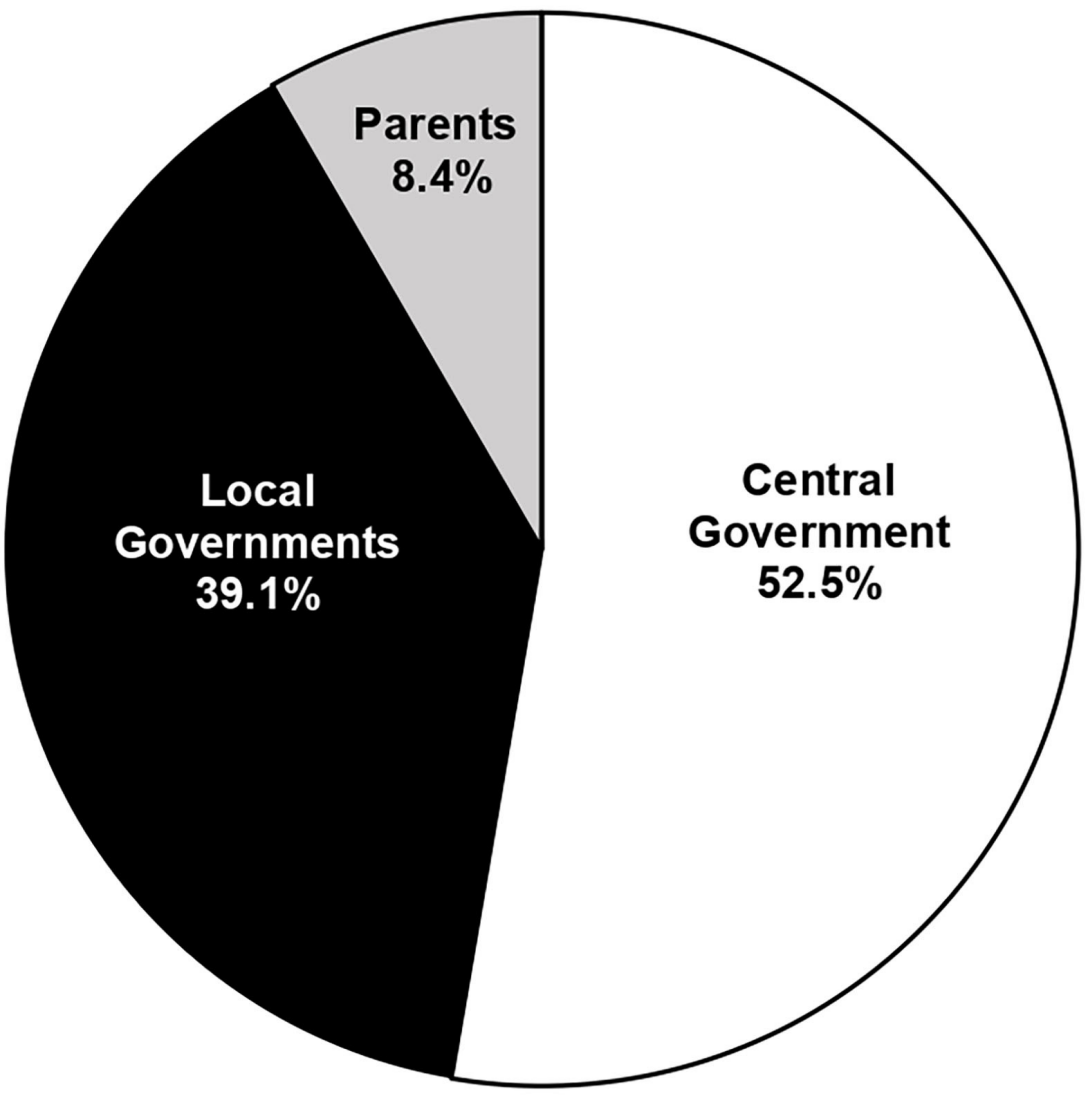
Pie chart illustrating of who should decide about school closures obtained from the Survey of Opinions of School Closures during the Current COVID-19.

Most respondents (74.2%) indicated they were parents (*n* = 6,457); 1,494 were teachers (17.2%); 377 were students (4.3%); 4.3% responded “none of the above”. Table 2 shows the responses to the survey grouped by all participants, inner and outer regional municipalities, and according to SES scores ≤40 and >41. Respondents overwhelmingly agreed that the government should be allowed to make the decision about school closures (91.6%). 52.5% felt the decision should be made by the central government and 39.1% felt it should be a local government decision. Only 730 participants (8.4%) felt parents should be allowed to make the choice about school attendance (χ^2^ = 4.011, *p* = 0.001). Figure 3 shows the distribution of responses to the three statements in the survey for all participants. There were no significant differences in responses on school closures between respondents inside and outside municipalities (χ^2^ = 4.184, *p* = 0.123) or by SES scores (χ^2^ = 3.93, *p* = 0.14) (Table 2).

**Table 2:** Responses to the SOSC-COVID-19 survey about initiating school closures by group: all respondents, regional municipalities, and SES scores above or below 40.

**Group**	**Closure initiated by government**		**No closure**	**χ^2^**	***p***
	**Centrally**	**Locally**	**Parental choice**		
	***n* (%)**	***n* (%)**	***n* (%)**		
All respondents (*N* = 8,703)	4,573 (52.5%)	3,400 (39.1%)	730 (8.4%)	4.011	0.001
**Regional municipalities**
Inner (*N* = 5,103)	2,701 (52.9%)	1,955 (38.3%)	447 (8.8%)	4.184	0.123
Outer (*N* = 3,600)	1,872 (52.0%)	1,445 (40.1%)	283 (7.9%)		
**SES scores**
≤ 40 (*N* = 711)	350 (49.2%)	302 (425%)	59 (8.3%)	3.93	0.14
>41 (*N* = 7,992)	4,223 (52.8%)	3,098 (38.8%)	671 (8.4%)		

### Qualitative Survey Results

After reading through all responses voiced in the open-ended question, most opinions involved concerns about how closures affected students' health, economic impacts to families, and why they did or did not support school closures. Categories, descriptions, and opinions are summarized in Table 3.

**Table 3:** Summary of feedback: categories, description, and opinions.

**Category**	**Description**	**Opinions**
1. Students' health	Risks of COVID-19 infections for children if schools remain open.	• Schools should be closed immediately. • Children would be safer not going to schools.
2. Economic concerns	School closures would impact the family economics.	• Families needed financial support from governments. • Children needed online learning support from the governments.
3. Reasons for decisions	Support of government closures	• Standards to close schools should be set by the central government. • Decisions to close schools should be at the local level because there are differences among school districts.
	Support for parental choice	• Parents should be allowed to decide. It is not proper to close the schools nationwide. • Every family situation is different, let parents make the decision to suspend school attendance.

#### Concerns About Students' Health

Many respondents mentioned they were worried children would be infected with the virus if the schools were not closed. One parent from the Changhua district wrote*, “New cases of COVID-19 increased rapidly in the last several days, and it will be too late if the school is not closed now. The students can go back to school when the pandemic subsides.”* Both teachers and parents supported school closures because they were concerned COVID-19 would be transmitted during classroom sessions or when students were eating without masks. A Taoyuan teacher wrote, “*Children will not tolerate wearing masks in hot weather and will be at risk of infection. We should avoid gathering in classrooms to reduce the risk of infection.”* A parent from Taichung City said, “*Children spend a long time in school. They take off their masks when eating lunch, which will increase the risk of infection.”*

Parents also worried about infection during transportation to schools and the sequelae of CPVID-19. A New Taipei parent wrote, “*Some students take the bus and Taipei Mass Rapid Transit (MRT), and they could be infected by COVID-19-infected classmates. It would be good to suspend classes as soon as possible.”* A Taichung parent said, “*Children can develop severe pulmonary fibrosis from a COVID-19 infection, and then they will have no future at all! Please suspend classes as soon as possible!”*

#### Economic Impacts

School closures carry high social and economic costs for communities. Employed parents are more likely to miss work when schools close to take care of their children. A Taichung parent wrote, “*Not every parent can take care of children during school closures. There should be supporting measures.”* A Changhua parent wrote, “*Schools should provide help to children without support, because not every family can take care of children during school closures, and this will cause problems.”*

Parents from areas with SES scores > 41 had concerns about the economic impact and challenges that low-income families (≤40) would face, which was expressed by a parent from New Taipei who wrote, “*Please provide more support to low-income families. If the parents take care of children and cannot go to work during school closures, they will lose their jobs and have no income.”* A Single parent from an area with a low SES score (Hualien County) said, “*Some single-parent families cannot provide computers or smartphones. It is not good for children to be alone at home during school closures. Instead, it causes social problems.”* A Taipei parent, who did not support nationwide school closures, was opposed due to concern about families for whom online teaching equipment was not affordable.

#### Reasons for Who Should Make the Decision About School Closures

Most respondents believed school closures should be decided by the central government because of the nationwide impact of the COVID-19 outbreak. A teacher from Hsinchu County wrote, “*A decision by either the central or local government to close schools is acceptable.”* A parent from Kaohsiung City wrote, “*There should be a unified standard by the central government. There will be inconsistent actions if school closures are not announced by the central government.”* However, a parent in Changhua County commented, “*The central government should provide standards of school closures, and the local government should make decisions according to the standards*.” Parents from the inner municipality of Taipei City (SES > 41) and the outer municipality of Yunlin County (SES ≤ 40) reported school closures should be decided by the local government because they were better able to address issues specific to each region, whereas the central government had multiple interests to juggle. One parent from Yunlin County said, “*It would be too late if the school closures were decided by the central government. The chief of the local government can judge and decide when to close the school in time* [to halt the spread of the virus]*.”*

The reason respondents believed parents should make the decision was explained by a teacher from the inner municipality of Taoyuan City who wrote, “*Every family situation is different”*. A parent from the outer municipality of Yilan County wrote, “*Even if school closures are not announced by the government, the parents should make the decision themselves. Do not overthink the situation.”*

### School Closures and Viral Transmission Among Students

Nationwide school closures are useful in preventing the spread of COVID-19 among students. MOE announced the closing of all schools on May 18, 2021. On May 19, 2021, nationwide level 3 epidemic prevention and control measures were implemented (Supplementary Table S1), without the need to initiate a nationwide full lockdown.

**Figure 4:**
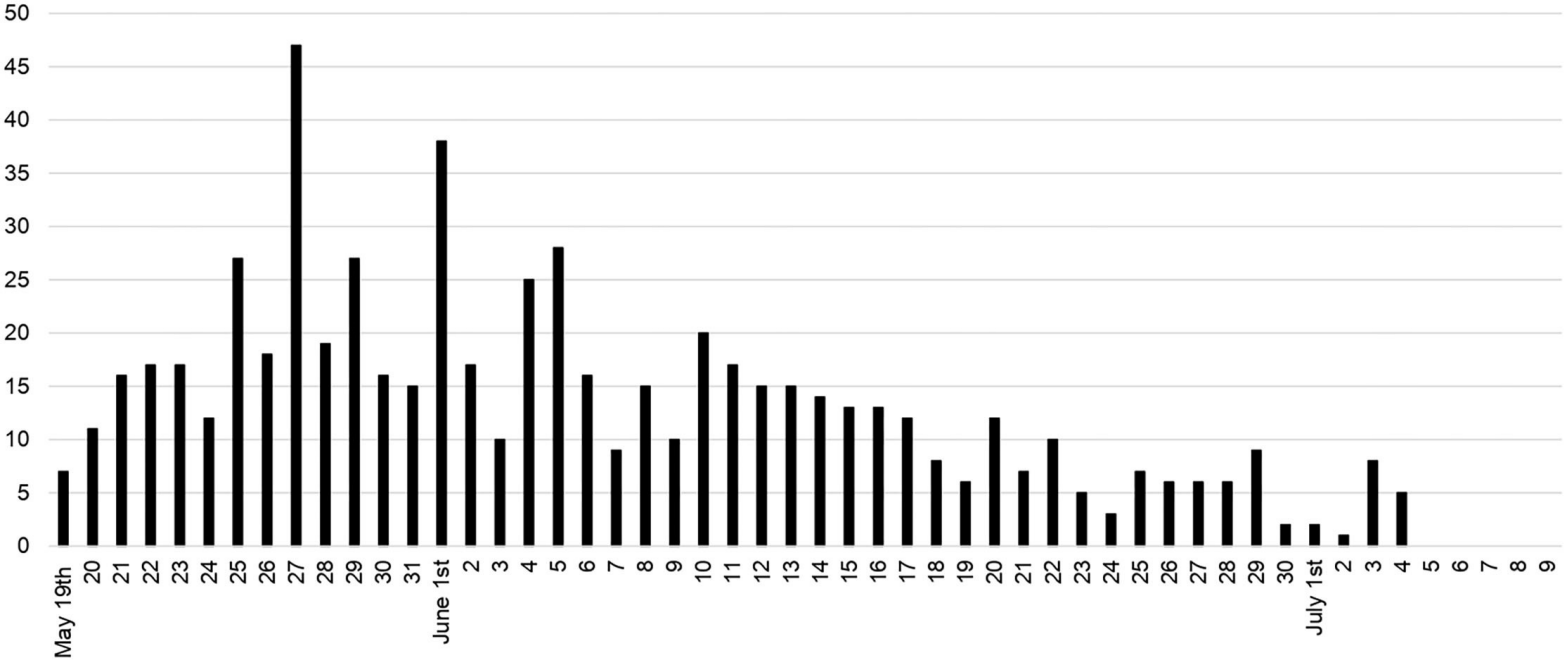
Number of new daily cases of COVID-19 among students in Taiwan following closures of all schools in Taiwan (Ministry of Education).

The initiation of Level 3 prevention and school closures began while the surge was increasing and there was an initial increase in newly diagnosed cases in Taiwan. However, cases began to decline significantly ~21 days later, as shown in Figure 2 (around June 6, 2021). The impact of school closures on viral transmission also benefited students. Cases of newly diagnosed COVID-19 among students initially increased from a rate of more than 10 cases per day to 47 cases on May 27. However, by June 6, 2021, new cases hovered around 15 per day with no new cases reported after July 5, 50 days after school closures (Figure 4). Most students who contracted COVID-19 were university students (205 cases, 30.7%, from April 20 to July 9).

## Discussion

Although the initiation of school closures to suppress transmission of COVID-19 impacts all families, they tend to have the greatest effect on families with low-income (9). However, in our study, respondents in areas with SES scores ≤ 40, an indicator of low-income, were as equally supportive of school closures as respondents from areas scoring >41. This suggests concern for reducing transmission and the health of the student population outweighed other considerations of respondents, regardless of economic status, thus prompting a call for school closures. Though nationwide school closures cannot stop transmission within families, this intervention can reduce transmission among students during COVID-19 outbreaks, which could have an indirect effect on prevention of transmission from children to families.

### Decisions About School Closures

Nearly all respondents indicated that school closures should be announced by central or local governments. The anonymous feedback indicated both parents and teachers supported giving government control of this decision because of the crucial nature of the COVID-19 outbreak and concern about students' health. A report proposed the implementation of a set of public, comprehensible, and data-driven criteria for school closures during the COVID-19 pandemic (9). The most common measures used are new case rates and test positivity rates, primarily at the county level in the United States (10). Taiwan implemented measures for school closures in local areas but did not have nationwide criteria for school closures (11). Thus, criteria for nationwide school closures should be implemented to avoid unplanned school closures during outbreaks.

### Nationwide School Closures Were Useful Interventions for COVID-19 Transmission in Schools

Many students infected by classmates or friends in the first few days of the outbreak went on to infect family members and others who contacted them (12). In one notable case that led to a cluster of outbreaks was one unknowingly infected individual transmitting COVID-19 not only to a group of friends who sang together at a karaoke parlor, but also the transmission of the virus to students in an adjacent room (13). These infected university students subsequently passed the virus to their roommates in university dormitories and their families, with a total of 9 individuals ultimately testing positive.

Following school closures, in combination with nationwide Level 3 epidemic prevention and control measures, new COVID cases and deaths decreased (Figure 2) and control measures were reduced to Level 2 on July 27, 2021. Data from MOE indicated the closing of schools on May 18, 2021, coincided with a reduction in the number of new cases of COVID-19 among students to zero 50 days after school closures. The comparison of the decline in COVID-19 cases among the total population of Taiwan (Figure 2) with COVID-19 cases among students over the same 50-day period (Figure 4), suggests the nationwide school closures had the greatest benefit for preventing transmission among students.

Several theoretical reasons could explain why school closures might be less effective for preventing the spread of COVID-19 compared with previous influenza outbreaks. Children contribute more to influenza transmission than do adults (14), but transmission in schools was low or absent during the previous coronavirus (SARS) outbreaks (15). It was reported school closures is predicted to be insufficient to mitigate (never mind suppress) the COVID-19 pandemic in isolation (16), there is no strong evidence available for the effectiveness of school closures for COVID-19 (17). Children appear to represent a lower proportion of COVID cases than would be expected for the size of their population, however, it might be due to children largely remaining asymptomatic or having a mild form of the disease (18). Children who contracted COVID-19 in school can easily pass the virus to other children as well as to adults. A granddaughter returned to Tainan from New Taipei, and infected her grandmother in Tainan (19). Data from Taiwan support our findings that the implementation of nationwide school closures further contributes to prevention of infection among students and lowering the risk of infection to families.

### Additional Measures to Suppress the Spread of COVID-19

The combination of preventive measures implemented in Taiwan suppressed the wave of COVID-19 transmission in May 2021, even as Australia, Vietnam, and Singapore were struggling with an uptick of the virus at the same time. These measures included strict border controls, close health monitoring, and quarantine measures for people entering Taiwan (20). Second, Taiwan doubled down on longstanding strategies of masking, quarantine measures, and contact tracing, and provided quarantine facilities, which significantly reduced transmission of the virus within families. Contact tracers leveraged activities by maintaining written records or canning a QR code provided by an app from their phones. Third, authorities banned indoor dining in the early days of the outbreak.

### Public Health Interventions and Effective Strategies Are Necessary to Help Parenting Difficulties

The feedback from parents about concerns for children's health and economic problems, including availability of online learning support, are similar to reports from parents in the United States, who worried about the impact of closures on their children's daily routines, the spread of COVID-19, and demands of online schooling (21). Parents in the United States also reported high levels of depression, anxiety, parental burnout, and increased negative emotions, such as anger and worry (22). Our findings provide additional confirmation that school closures during COVID-19 are stressful for parents. Public health interventions should address parenting-specific stressors and effective strategies for managing parenting difficulties to mitigate their deleterious impact.

### Limitations

Our findings have some limitations. The critical surge in COVID-19 cases prompted the survey to be rapidly designed and processed on May 17, 2021. Therefore, the validity and reliability of the survey was not analyzed. Although the survey results were useful in transmitting the message to MOE that 91.6% of respondents wanted schools closed immediately, the survey lacked demographic information. A follow-up survey with demographic information will be conducted in the future. Only 3.6% participants were from Taipei and New Taipei, where school closures had already been announced. Although 85–90% of Taiwanese over 16 years of age use mobile phones and have access to the Internet, few respondents (8.2%) were from low-income areas of Taiwan and few of these respondents provided any personal feedback. A lack of Internet access would limit receiving information through social media channels as well as the ability to complete the online survey in the short period of time.

## Conclusions

The SOSC-COVID-19 was disseminated in response to the desire of parents to close schools. The survey results were sent to the MOE for reference; however, the decision was made prior to the MOE receiving the survey results. Although school closures addressed the concerns expressed by parents in the survey's feedback, no information is available as to how the closures impacted learning loss of children and economic stability of families, which should be examined with future studies.

School closures carry high social and economic costs for communities. Their impact is particularly severe for the most vulnerable and marginalized children and their families (23). Schools are essential for children's learning, health, safety and wellbeing (24), and are particularly vital for children primary school age children (25). The consequences of school closures could be felt for decades and are contributing to even wider inequality, particularly for girls (25). Working parents are more likely to miss work when schools close to take care of their children, which results in wage loss and possibly job loss (23). Future research should collect information to estimate the scale of learning loss and economic harms during school lockdowns moving forward.

## Data Availability Statement

The raw data supporting the conclusions of this article will be made available by the authors, without undue reservation.

## Ethics Statement

The studies involving human participants were reviewed and approved by Ethics Committee of Taipei Hospital, Ministry of Health and Welfare. Written informed consent for participation was not required for this study in accordance with the national legislation and the institutional requirements.

## Author Contributions

T-YH designed and disseminated the survey. K-YC collected the data and processed the analyses. WC conceived the study, wrote the manuscript, and took primary responsibility for communication with the journal and editorial office throughout the submission, peer review, and publication processes. All authors contributed to the article and approved the submitted version.

## Conflict of Interest

T-YH was a volunteer of National Alliance of Parents Organization. The remaining authors declare that the research was conducted in the absence of any commercial or financial relationships that could be construed as a potential conflict of interest.

## Publisher's Note

All claims expressed in this article are solely those of the authors and do not necessarily represent those of their affiliated organizations, or those of the publisher, the editors and the reviewers. Any product that may be evaluated in this article, or claim that may be made by its manufacturer, is not guaranteed or endorsed by the publisher.
