# Supplementary material for: Survey Responses of School Closures During the COVID-19 Outbreak in Taiwan
Source: Front Public Health. 2022 Mar 16;10:726924. doi: 10.3389/fpubh.2022.726924 (PMC8966014; doi:10.3389/fpubh.2022.726924)
Supplement: Supplementary file 1 [file Table_1.DOCX]

**Supplement table 1S Level 3 epidemic prevention and control measures (**Source: Taiwan Centers for Disease control)

| **Disease prevention and control implemented under the Level 3 epidemic alert** |
| --- |
| A. Members of the public must wear a mask all the time when going out. Once a violation of the rule is found, penalties will be imposed without prior warnings.  B. Leisure and entertainment venues that have been ordered to close must not continue operations, and frequent inspections will be conducted at leisure and entertainment venues. For those failing to abide by the rule, the business owner, staff, customers and participants of the gathering at the site will face their respective penalty.  C. Food and beverage vendors should only offer takeout services, and stores and supermarkets should bolster crow control measures. Members of the public are urged to reduce the frequency of visiting supermarkets/stores and buy more at one go.  D. Wedding banquets and public memorial ceremonies of funerals are banned.  E. Religious gatherings or events are fully suspended; places of worship are temporarily closed to the public.  F. Closure of Leisure and entertainment venues, sports competition and exhibition venues and educational facilities.  G. All family or social gatherings involving over 5 people indoors or 10 people outdoors (those living together not included) are suspended; avoid unnecessary movement, activities or gatherings.  H. Self-health monitoring; those with symptoms should seek medical attention.  I. Business venues and places where personal business is conducted should implement crowd and flow control and enforce the wearing of masks and social distancing.  J. Workplaces should follow the epidemic prevention rules in the Guidelines for Enterprise Planning of Business Continuity in Response to the Coronavirus Disease 2019, and put individual and work place health management mechanisms into action, as well as measures to maintain business continuity (like working in multiple offices, distance working and flexible working hours).  K. Enhanced disinfection of public places and public transport. |
